# Effect of virtual reality and haptic feedback on upper extremity function and functional independence in children with hemiplegic cerebral palsy: a research protocol

**DOI:** 10.11604/pamj.2022.41.155.32475

**Published:** 2022-02-22

**Authors:** Chanan Goyal, Vishnu Vardhan, Waqar Naqvi, Sakshi Arora

**Affiliations:** 1 Government Physiotherapy College, Raipur, India,; 2 Ravi Nair Physiotherapy College, Datta Meghe Institute of Medical Sciences, Wardha, India,; 3 Department of Cardiorespiratory Physiotherapy, Ravi Nair Physiotherapy College, Datta Meghe Institute of Medical Sciences, Wardha, India,; 4 Department of Community Physiotherapy, Ravi Nair Physiotherapy College, Datta Meghe Institute of Medical Sciences, Wardha, India

**Keywords:** Hemiplegic cerebral palsy, physiotherapy, virtual reality, haptic feedback, functional independence

## Abstract

Hemiplegic cerebral palsy (CP) is a subcategory of CP which is characterized by sensory motor deficits primarily on one side of the body that adversely affects functionality. Virtual reality (VR) systems have been advanced in the recent past for the use in rehabilitation of patients with neurological conditions. Virtual reality has an inherent motivational component that provides the much-needed compliance for active participation by children. The rationale of the proposed study is to investigate the effect of VR and haptic feedback for improvement of upper extremity function of children with hemiplegic cerebral palsy. This comparative experimental study will be recruiting 36 children with hemiplegic CP and will be treating them by VR and haptic feedback along with conventional physiotherapy in group A and by conventional physiotherapy only in group B. The children will undergo the treatment for six weeks (five days/week) with each session extending for 60 minutes/day. The primary outcome measures including `nine-hole peg test´ (9HPT) and `box and block test´ (BBT) will assess the manual dexterity and secondary outcome measures including `ABILHAND-kids´ and `WeeFIM (self-care)´ will assess the functional independence that are hypothesized to be gained by haptic enhanced VR intervention when combined with the conventional therapy.

## Introduction

**Background and rationale:** cerebral palsy (CP) in India has a prevalence of 2.95 per 1000 children (95% CI 2.03-3.88) [1]. Hemiplegic CP is a subcategory that accounts for a substantial number of all cases [2]. It is characterized by sensory motor deficits, primarily on one side of the body, that adversely affects functionality. Approximately 50% of children with hemiplegic CP have greater impairment in the upper extremity, particularly in the hand, as compared to the lower extremity [3]. Preferential hand usage of the non-paretic hand often results in the neglect of the paretic hand that adversely affect gross motor function classification system (GMFCS) and manual ability classification system (MACS) scores. Technologically advanced virtual reality (VR) systems have been used in the recent past in rehabilitation of patients with neurological conditions. Although numerous studies report promising results in adults with stroke [4,5], a limited number of studies have been undertaken that combine the use of VR and haptic feedback for children with infantile hemiplegia. Ravi *et al*. concluded that there is a need for research to establish the place of VR rehabilitation for children with CP [6]. Virtual reality has an inherent motivational component that provides the much-needed compliance for active participation by children. The inner drive to play VR based games that provide a real-world experience, is almost invariably present in school-aged children. This can be tapped for obtaining therapeutic benefits. Haptic feedback or force feedback enhances the fine-tuning of desired motor responses [7]. The use of paretic hand during the session may lead to the carry over for the functional tasks that can be gauged using appropriate outcome measures. The rationale of the proposed study is to investigate the effect of VR and haptic feedback for improvement of upper extremity function of children with hemiplegic cerebral palsy. The parents of children with infantile hemiplegia have a common primary concern of less or no functional use of paretic hand. Activities like reaching out, grasping, carrying and releasing by the affected upper limb are often inadequate. They need assistance in activities of daily living. Parents mention that children need repeated prompts for the use of paretic hand and that the children refrain from taking initiative to put the affected hand to function. This study aims to utilize the appeal in VR gaming systems and haptic feedback for bringing out the children´s inherent potential to use their paretic upper extremity and objectively record its effectiveness in improving function.

Virtual reality has an inherent motivational component that provides the much-needed compliance for active participation by children. The inner drive to play VR based games that provide a real-world experience, is almost invariably present in school-aged children. This can be tapped for obtaining therapeutic benefits. Haptic feedback or force feedback enhances the fine-tuning of desired motor responses [7]. The use of paretic hand during the session may lead to the carry over for the functional tasks that can be gauged using appropriate outcome measures. The rationale of the proposed study is to investigate the effect of VR and haptic feedback for improvement of upper extremity function of children with hemiplegic cerebral palsy. The parents of children with infantile hemiplegia have a common primary concern of less or no functional use of paretic hand. Activities like reaching out, grasping, carrying and releasing by the affected upper limb are often inadequate. They need assistance in activities of daily living. Parents mention that children need repeated prompts for the use of paretic hand and that the children refrain from taking initiative to put the affected hand to function. This study aims to utilize the appeal in VR gaming systems and haptic feedback for bringing out the children´s inherent potential to use their paretic upper extremity and objectively record its effectiveness in improving function.

**Objectives:** objectives of the study are to find the effectiveness and impact of VR and haptic feedback along with conventional physiotherapy (PT) for improving the upper extremity function, functional independence in children with hemiplegic cerebral palsy. Also, we will be evaluating the difference between improvement of upper extremity function by VR with haptic feedback along with conventional physiotherapy and by conventional physiotherapy only.

## Methods

**Figure 1:**
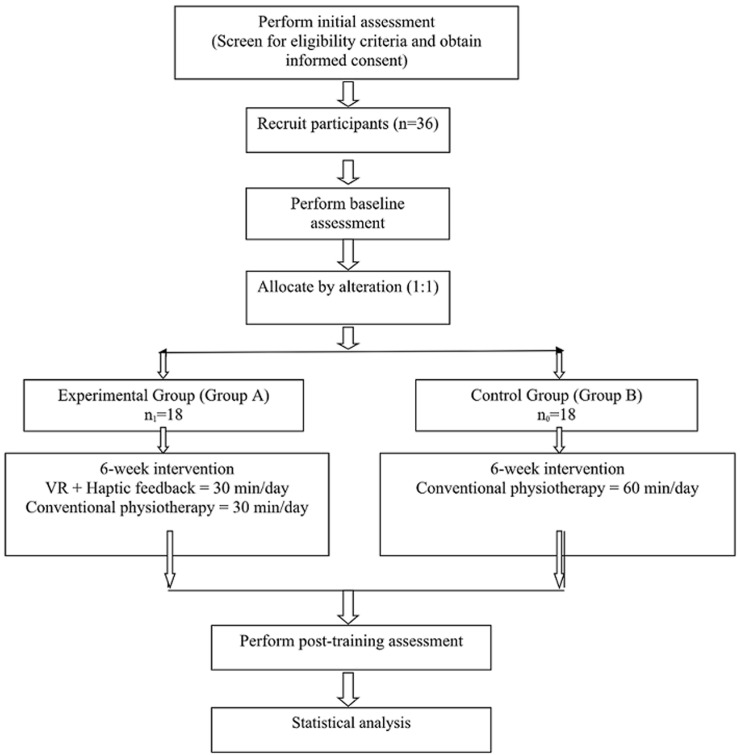
shows the study procedure in the form of a flow chart

The enrollment, intervention, and assessment of this study will be followed as recommended by standard protocol items recommendation for intervention trials (SPIRIT), 2013 as illustrated in Table 1 and Figure 1 [8].

**Table 1:** schedule of enrolment, intervention and assessment where t1 is enrolment and pre-intervention assessment

	Study period			
	Enrolment	Allocation	Post-Allocation	
Timepoint	-t1	0	Intervention (t1-t10)	Post-test (t11)
**Enrolmen**t				
Eligibility screen	X			
Informed consent	X			
Allocation		X		
**Intervention**				
VR and haptic feedback + Conventional PT			X	
Conventional PT			X	
**Assessment**				
**Baseline variables**				
GMFCS, MACS	X			
**Outcome measures**				
9HPT, BBT, ABILHAND-Kids, WeeFIM (self-care)	X			X

t1-t10: duration of intervention; t11: post intervention assessment; VR: virtual reality; PT: physiotherapy; GMFCS: gross motor function classification system; MACS: manual ability classification system; 9HPT: nine hold peg test; BBT: box and blocks test; WeeFIM: functional independence measure for children

**Study setting:** this trial will be conducted at Neurosciences Center, Department of Neurosurgery and Neurology, Neurorehabilitation Division of Acharya Vinoba Bhave Rural Hospital, Datta Meghe Institute of Medical Sciences, Sawangi (Meghe), Wardha, Maharashtra, India. The children referred for physiotherapy intervention at the Neurorehabilitation Division will be screened for eligibility criteria.

**Eligibility criteria:** the inclusion criteria will comprise school aged children between 6 and 12 years diagnosed with hemiplegic CP, mild or no difficulty understanding commands, ability to communicate age appropriately or with some difficulty (but a new listener can understand), no uncorrected vision, hearing without the need of a hearing aid, manual ability classification system (MACS) level I-III, gross motor function classification system (GMFCS) level I-III, and willingness to participate. However, the exclusion criteria will be mixed or other types of CP, cognitive impairment to an extent that defers understanding of commands, epilepsy, severe visual and/or auditory deficit, history of surgical procedures in last 6 months, history of botox treatment in last 3 months and enrollment in other clinical trials.

**Figure 2:**
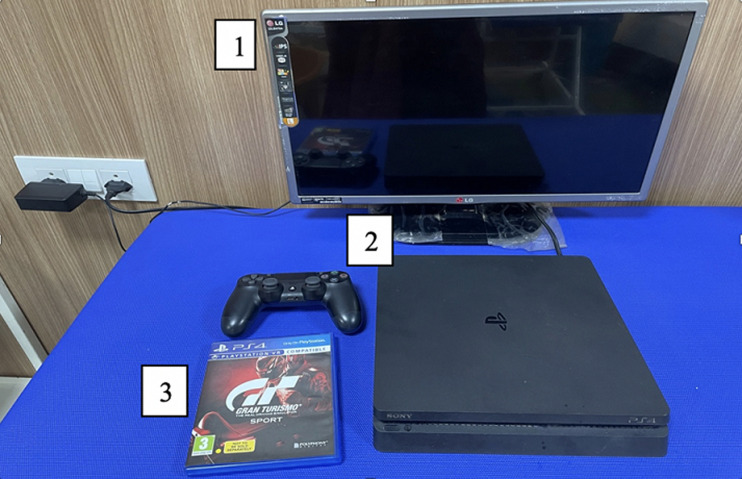
material that will be used for providing virtual reality and haptic feedback-based intervention for participants in the experimental group, including television screen (1), playstation 4 that provides virtual reality-based gaming with haptic feedback enhanced hand controls (2), DVD of a game named Gran Turismo- the real driving simulator (3)

**Intervention:** the participants in the experimental group (group A) will undergo 30 minutes of conventional physiotherapy including activities for strengthening of weak muscles like wrist extensors and forearm supinators, stretching of tight muscles like wrist flexors and forearm pronators, weight-bearing activities like push-ups and quadripod position along with 30 minutes of VR and haptic feedback-based training of upper extremity by playing a serious game with real life-like experience and force feedback. Material for VR and haptic feedback intervention is shown in Figure 2. The participants in the control group (group B) will receive 60 minutes of conventional physiotherapy including activities for strengthening of weak muscles like wrist extensors and forearm supinators, stretching of tight muscles like wrist flexors and forearm pronators, weight-bearing activities like push-ups and quadripod position, along with reach, grasp, carry and release activities and fine motor activities for hand function training using pegboards. Post-intervention data will be collected for both groups using primary and secondary outcome measures.

### Outcomes

**Figure 3:**
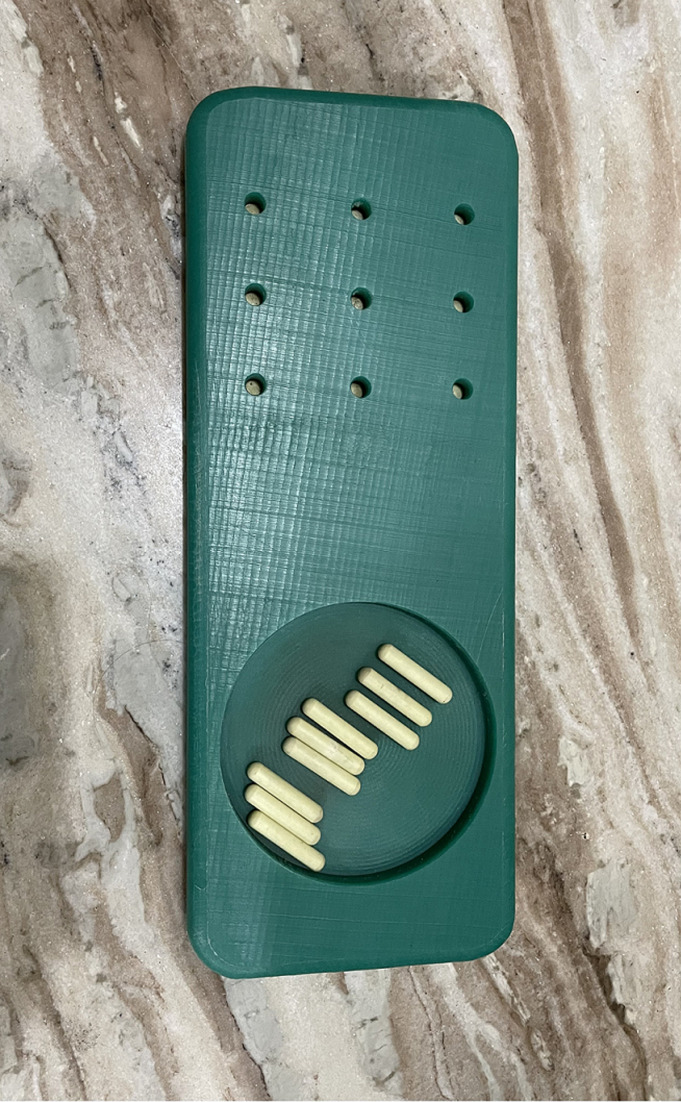
shows nine pegs with nine-hole peg board that will be used for 9HPT

**Figure 4:**
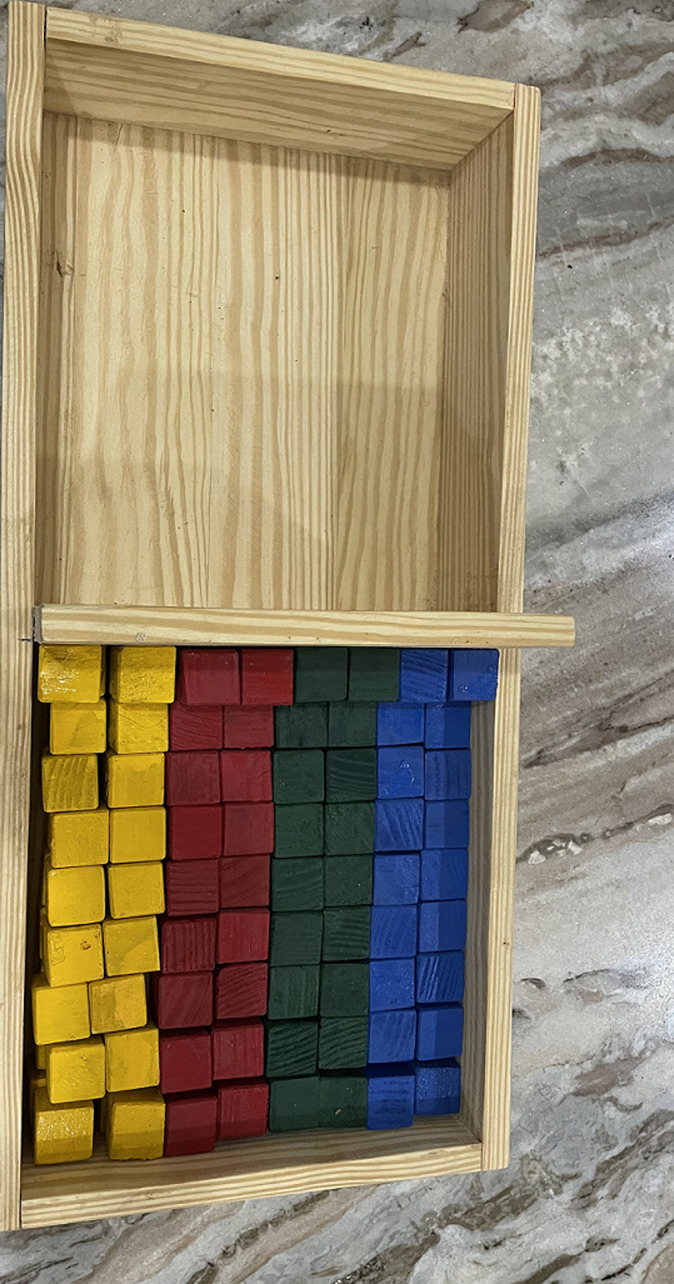
shows 150 blocks with a partitioned box that will be used for BBT

**Primary outcome measures:** nine hole peg test (9HPT) will be used to evaluate fine motor function. Moderately high test-retest reliability and high interrater agreement was demonstrated by Smith *et al*. [9]. Correlations between the scores on the 9HPT and purdue pegboard test reveal sufficient concurrent validity of these measures, and a significant difference in test scores between regular and special education groups provided further indication of construct validity. Thus, 9HPT is an effective tool for estimating the fine motor dexterity of school-aged children [9]. The board with pegs for 9HPT is shown in Figure 3 box and blocks test (BBT) will be used as a test of unilateral gross manual dexterity. It is administered by asking the subject to one-by-one move, the maximum number of blocks from one compartment of a box to another of equal size, within 60 seconds. The test showed very high inter-rater, test-retest reliability and excellent construct validity [10] BBT kit is shown in Figure 4.

**Secondary outcome measures:** ABILHAND-Kids will be used to evaluate upper extremity function. It is a 21-item functional ordinal scale specifically developed to measure manual ability in children with CP. Its measurement precision is considered highly adequate for clinical practice. Parents are requested to fill the questionnaire on the basis of their perception of the level of difficulty that a child faces during the performance of each activity in the list on a three-tier scale of `impossible´, `difficult´, `easy´ [11]. The linear measures obtained by the Rasch model can be used to make a comparison quantitatively regarding the manual ability of children with CP [12]. The test has shown excellent test-retest reliability as well as internal consistency [11]. It has excellent convergent validity with MACS [13]. WeeFIM (self-care section) will be used for evaluating functional independence. Functional independence measure for children is also known as WeeFIM. It can be used to estimate the `burden of care´ for children with developmental disabilities between 6 months to 21 years of age [14]. The performance of task is categorized as `dependent´ if the score is between 1-5 and is categorized as `independent´ if the score is 6 or 7. Score 1 indicates total assistance, score 2 indicates maximal assistance, score 3 indicates moderate assistance, 4 indicates minimal assistance, score 5 indicates supervision or set-up, score 6 indicates modified independence that includes an assistive device usage or not completing the task in a timely or safe manner and score 7 indicates complete independence i.e. the child completes the task without using a device [14]. The test-retest reliability is excellent for children with disabilities [15].

**Sample size:** sample size of 36 was calculated with 90% of confidence interval considering the dropouts, with 18 participants in each group by using G*Power software. A convenient sampling technique will be used.

**Recruitment and allocation:** potential participants will be screened for eligibility criteria and after obtaining well-informed written consent of the parent(s) or legal guardian, the child will be recruited as a participant. The participants will be distributed in 1: 1 manner into experimental group/group A (VR and haptic feedback in addition to the conventional physiotherapy) and control group/group B (conventional physiotherapy only).

**Data collection:** pre-intervention data will be collected using predetermined primary and secondary outcome measures. The training would be carried out for 6 weeks (5 days a week). The duration of each session would be 60 minutes. Virtual reality and the haptic feedback with conventional physiotherapy, and conventional physiotherapy alone are independent variables. Upper extremity function and functional independence are dependent variables. Data collected will be noted down and will be placed in a tabular format.

**Statistical methods:** after consultation with the statistician, statistical analysis will be done by using descriptive and inferential statistics using t-test, Wilcoxon signed-rank test and Mann-Whitney U test. Software SPSS 27.0 version and GraphPad prism 7.0 version will be used in the analysis. The changes in the outcome measures brought about after the course of 6 weeks of intervention will be analyzed by mixed-effects linear models across `time´ (pre-intervention vs post-intervention) and `group´ (experimental vs control). The age of participants and baseline scores of outcome measures between the two groups will be compared using t-tests. A p-value of less than 0.05 will be considered as level of significance. If subjects are lost to follow-up, an intention-to-treat analysis will be carried out. The results will be interpreted to note any significant statistical and/or clinical differences. The results will be accounted and documented as per the CONSORT guidelines.

**Trial design:** this is an interventional, non-randomized, active controlled, superiority trial with allocation ratio of 1: 1 in experimental group and control group.

**Ethics approval:** the research study was ethically approved by the Institutional Ethics Committee, Datta Meghe Institute of Medical Sciences on 29^th^ January 2021 with reference number DMIMS (DU)/IEC/2020-21/131. The Doctoral Research Committee approved it on 9^th^ April, 2021. The research protocol was submitted to Clinical Trials Registry of India on 16^th^ November, 2021 with reference number REF/2021/11/048970. The trial was registered in Clinical Trials Registry of India (CTRI) on 23^rd^ December 2021 with the registration number CTRI/2021/12/038861.

## Discussion

Virtual reality (VR) has emerged as a promising intervention for improving motor function in children with CP in a few previous studies, though groups were heterogeneous with a small sample size [6,16,17]. The proposed study aims to estimate the efficacy of haptic enhanced VR with conventional physiotherapy compared to only conventional physiotherapy in improving upper extremity (UE) function and functional independence in school-aged children with hemiplegic CP. Diminished motor control of the affected upper extremity leads to neglect and learned disuse by the child that hampers its sensory-motor development [18]. The vicious cycle of weakness and disuse needs to be broken for improving function, which is usually a challenging task for children with infantile hemiplegia who are otherwise ambulatory. Gross and fine motor dexterity, as well as the use of paretic hand in bimanual activities, is the major area of concern of most parents. Though constraint induced movement therapy has been considered effective in the management of infantile hemiplegia, it lacks the much-needed motivation for the child that limits the number of repetitions of movements and the long-term compliance [19].

On the other hand, the VR-based rehabilitation is designed to offer therapy is characterized by repetitive task-specific movements that include abduction and opposition of the thumb, which is often difficult for children with hemiplegic CP on the affected side. Haptic feedback finely tunes the motor response within a virtual environment, and the children are expected to look forward for such enjoyable sessions to tap their high neuroblastic potential. Play station is a motivational training tool for children with CP and has the potential to improve UE function [20]. The primary outcome measures will assess the manual dexterity (9HPT and BBT) and secondary outcome measures (ABILHAND-Kids and WeeFIM) will assess the functional independence that are hypothesized to be gained by haptic enhanced VR intervention when combined with the conventional therapy. The advantages of the haptic enhanced VR study include the customized approach towards the rehabilitation protocol and the responsiveness of the VR system during the intervention. To avoid any disparity between the two groups, the total rehabilitation time will be the same for both groups. The results of the study will aid the children with unilateral CP in the future by providing a fun way of utilizing the advanced technology of rehabilitation.

**Limitations:** this study will not focus on participation of the child in the society in context of International Classification of Functioning, Disability, and Health (ICF). Long term follow up to observe maintenance or regression of the possible effects will not be carried out. Investigator is not blinded.

**Probable implications:** motor learning principles are well knit in context of VR along with force/tactile feedback that would help the children in learning skills related to manual dexterity. If the children show improvement in their hand function, burden of care on the caregivers will decrease as the child will become independent in activities of daily living. The study may pave the way for further advancement of technology for the use in younger children with infantile hemiplegia for early intervention.

## Conclusion

The interpretation of the results of the study will help to gain a better understanding of the utility of haptic enhanced VR based physiotherapy intervention in children with hemiplegic or unilateral CP.
